# Losses of human disease-associated genes in placental mammals

**DOI:** 10.1093/nargab/lqz012

**Published:** 2019-10-24

**Authors:** Virag Sharma, Michael Hiller

**Affiliations:** 1 Max Planck Institute of Molecular Cell Biology and Genetics, 01307 Dresden, Germany; 2 Max Planck Institute for the Physics of Complex Systems, 01187 Dresden, Germany; 3 Center for Systems Biology Dresden, 01307 Dresden, Germany

## Abstract

We systematically investigate whether losses of human disease-associated genes occurred in other mammals during evolution. We first show that genes lost in any of 62 non-human mammals generally have a lower degree of pleiotropy, and are highly depleted in essential and disease-associated genes. Despite this under-representation, we discovered multiple genes implicated in human disease that are truly lost in non-human mammals. In most cases, traits resembling human disease symptoms are present but not deleterious in gene-loss species, exemplified by losses of genes causing human eye or teeth disorders in poor-vision or enamel-less mammals. We also found widespread losses of *PCSK9* and *CETP* genes, where loss-of-function mutations in humans protect from atherosclerosis. Unexpectedly, we discovered losses of disease genes (*TYMP, TBX22, ABCG5, ABCG8, MEFV, CTSE*) where deleterious phenotypes do not manifest in the respective species. A remarkable example is the uric acid-degrading enzyme *UOX*, which we found to be inactivated in elephants and manatees. While *UOX* loss in hominoids led to high serum uric acid levels and a predisposition for gout, elephants and manatees exhibit low uric acid levels, suggesting alternative ways of metabolizing uric acid. Together, our results highlight numerous mammals that are ‘natural knockouts’ of human disease genes.

## INTRODUCTION

Natural selection purges mutations that have deleterious effects on fitness. This explains why mutations that are associated with human diseases tend to occur at positions that evolve under evolutionary constraint and this holds true for disease-associated variants located in both coding and non-coding genomic regions (1–4). These observations are exploited in medical genetics, where a common task is to rank a list of variants obtained by sequencing the exome or entire genome of a patient to identify the causal pathogenic mutation(s). Indeed, most computational methods that predict deleteriousness of human variants use evolutionary constraint as a powerful predictive factor (5–10).

Despite the utility of evolutionary sequence constraint in predicting pathogenic variants, several studies discovered that some human disease-associated amino acid changes actually occur as wild-type alleles in other species ranging from Neanderthals and chimpanzees to non-primate mammals (11–14). Similar findings have been reported in insects, where mutations that are deleterious in *Drosophila melanogaster* have been observed in other insects (15). Sequence and protein structure analysis provided evidence that disease-associated amino acid changes are permissible in other species because mutations at other sites in the same protein neutralize the effect of the deleterious mutation(s) and restore function (12,14,16,17). Thus, the effect of an amino acid mutation depends on the sequence context, which probably explains why the same mutation can be neutral in other species but leads to loss of protein function and disease in humans.

In addition to amino acid mutations, other disease-associated mutations cause loss-of-function by abolishing the production of a full-length protein. Such mutations include premature stop codon, frameshift or splice site mutations that inactivate the reading frame. While amino acid changes can be permissible in the context of other mutations in the same gene, it is rather unlikely that this also applies to mutations that completely inactivate a gene. One would therefore generally expect that gene-inactivating mutations in human disease-associated genes do not occur in other mammals. However, while the presence of human disease-associated amino acid changes in other species has been clearly established, the presence of inactivating mutations in orthologs of human disease-associated genes in non-human mammals has not been comprehensively investigated.

To investigate whether disease-associated genes can be inactivated in the course of mammalian evolution, we systematically analyzed genes that are lost in placental mammals. We show that genes lost in at least one of 62 non-human mammals are highly depleted in disease-associated genes and genes performing essential functions. Despite these expected depletions, we found multiple losses of human disease genes in non-human mammals where disease phenotypes are present but not deleterious. Unexpectedly, we also discovered several disease gene losses where the disease phenotypes do not appear to manifest in gene-loss species. For example, while the loss of the uric acid degrading *UOX* gene in human and related hominoids is implicated in increased serum uric acid levels and a predisposition to gout, we found that elephants and manatees exhibit low uric acid levels despite having lost the same gene. Overall, our results highlight numerous mammals that are ‘natural knockouts’ for genes implicated in human disease and show that even complete losses of disease-associated genes can occur in evolution.

## MATERIALS AND METHODS

### Gene losses in placental mammals

We used data from a previously developed approach that systematically detects gene-inactivating mutations such as stop-codon mutations, frameshifting insertions and deletions, mutations that disrupt splice sites (deviation from the donor GT/GC or acceptor AG), and the deletion or loss of entire exons or even entire genes (18). This approach integrates a number of filtering steps to overcome issues with genome assembly or alignment and addresses changes in the exon–intron structure of conserved genes. These steps include (i) distinguishing assembly gaps from real deletions (19), (ii) re-aligning coding exons with CESAR, a method that makes use of reading frame and splice site information to correct alignment ambiguities and evolutionary splice site shifts (20,21), (iii) discarding alignments to genomic regions encoding the paralog or processed pseudogene of the gene of interest and (iv) considering all principal APPRIS isoforms of a gene (22). The analyzed data are based on the human Ensembl gene annotation (version 90), APPRIS principle isoforms (Gencode version 26), and a whole genome alignment between the human hg38 genome assembly (reference) and the genome assemblies of other (query) placental mammals (23) (Supplementary Table S1). All detected gene-inactivating mutations were used to determine the maximum percentage of the reading frame that remains intact in a query species (18,24). A gene was classified as lost if <60% of the reading frame remained intact and if at least 20% of the exons exhibit inactivating mutations. An exception are single-exon genes, where we simply required at least two inactivating mutations.

### Large-scale characteristics of lost genes

We compared genes that are classified as lost in at least one of the 62 placental mammals to those that are not classified as lost in any mammal. We used Dollo parsimony to infer whether a gene loss likely happened in a single lineage (such as a shared loss between mouse and rat) or in independent lineages.

To assess the pleiotropy of groups of genes, we used the Mouse Genome Informatics (MGI) Phenotype ontology (25,26) that lists phenotypes observed in mouse gene knockouts, organized into hierarchical levels of distinct phenotypes. We reasoned that the degree of pleiotropy of a gene should positively correlate with the number of distinct knockout phenotypes. We downloaded the MGI table MGI_PhenoGenoMP.rpt that lists knockout phenotypes and propagated lower level phenotypic terms to higher level (parent) nodes using the graph structure given in MPheno_OBO.ontology. We only considered phenotypes observed in the knockout of a single gene and excluded the level 3 term ‘no abnormal phenotype detected’. The MGI table MGI_Gene_Model_Coord.rpt was used to convert mouse MGI gene identifiers to mouse Ensembl gene identifiers. One-to-one orthologous coding genes downloaded from Ensembl BioMart (27,28) were used to map mouse to human Ensembl gene identifiers. Then, we determined the number of knockout phenotypes per gene separately for phenotype levels 2, 3 and 4. A two-sided Wilcoxon rank-sum test was used to test whether the number of knockout phenotypes is significantly different between genes not classified as lost in any of the 62 mammals, genes classified as lost in one lineage, and genes lost in more than one independent lineage.

To further explore depletions or enrichments of lost genes, we determined how many of the lost and non-lost genes are disease genes, essential genes, lethal genes and dispensable genes. As disease genes, we used human disease-associated genes whose mouse knockout models the human disease. These genes were obtained from the MGI table MGI_OMIM.rpt (26). Essential genes are those that are required for viability of the haploid human cell lines KBM7 and HAP1 (29). We used the 1734 genes that are essential for both cell lines. Lethal genes are those that result in ‘prenatal lethality’ (MP:0002080) in a mouse knockout. Dispensable genes are those that result in no detectable abnormal phenotype in a mouse knockout (genes only annotated with the level 2 term MP:0002873 ‘normal phenotype’). A two-sided Fisher’s exact test was used to test for significant differences. To test for functional enrichments of genes that are lost in at least one placental mammal, we used the gProfiler (Version: r1709_e87_eg34) over-representation analysis (30).

### Validation of novel gene losses

We performed the following analyses to verify that the nine genes discussed in detail below are truly lost. First, we excluded the possibility that a functional copy of the lost gene exists in the genomes of species that presumably lost this gene. To this end, we manually inspected the genome alignment chains (31) between human and every gene-loss species in the UCSC genome browser (32). These chains were computed with alignment parameters that are sufficiently sensitive to even capture paralogs that duplicated before the split of mammals (23), and should therefore reveal functional gene copies if they exist. Second, we inspected the top-level alignment chain in a larger genomic locus to confirm that the remnants of the lost gene are located in a context of conserved gene order.

Third, we validated the correctness of all gene-inactivating mutations in every gene-loss species using two approaches. First, we investigated whether inactivating mutations are shared with related species. To this end, we obtained additional recently sequenced mammalian genomes from NCBI or from the UCSC genome browser (Supplementary Table S1), in addition to the genomes already contained in our whole-genome alignment (23). New genomes were aligned to the human hg38 assembly as described before (23). Then, we manually confirmed the presence of shared inactivation mutations. Second, for those mutations that are likely species-specific or mutations in mammals for which sister species genomes are not yet available, we validated inactivating mutations by unassembled DNA sequencing reads stored in the NCBI sequence read or trace archive, as previously described (33,34). Briefly, we extracted the genomic sequence 50 bp upstream and downstream of a mutation and aligned it against sequencing reads using the blastn web-interface in megablast mode. We required that the mutation is confirmed by at least five reads without support for the ancestral non-gene-inactivating allele. While no unassembled sequencing reads are available for the Tibetan antelope, preventing a validation of antelope-specific mutations, each of the four genes lost in this species (*TYMP, CTSE, PCSK9, CETP*) have mutations that are shared with sister species, showing that these genes are truly lost. Apart from the Tibetan antelope, all smaller inactivating mutations shown in Supplementary Figures S1 and S2 and S4–S9 are validated by either sequencing reads or by their presence in sister species genomes.

### Loss of the uric acid degradation pathway in elephants and manatees

It is known that hominoids have inactivating mutations in *UOX* and *URAH*, but *URAD* still encodes an intact reading frame and is annotated as a gene in human (35,36). Our gene loss data showed that elephants have lost *URAD*. Based on this observation, we manually inspected the other two genes in the uric acid degradation pathway (*Uox* and *Urah*) in elephant using the mouse 60-way genome alignment provided by UCSC (37). This revealed that elephant *UOX* has a stop codon in exon 3, which is supported by several raw Sanger sequencing reads stored in the NCBI trace archive. Since exon 3 was targeted by a reading frame disrupting neomycin cassette insertion in mouse to create a *Uox* null mutation (38), the stop codon in elephant exon 3 most likely results in a non-functional truncated UOX protein. Our analysis also revealed that the manatee *UOX* has a 4 bp deletion in exon 4 and a stop codon in exon 5 (both supported by sequencing reads). The inactivation of *UOX*, the key enzyme in the uric acid degradation pathway, suggests that elephant and manatees have lost this pathway.

## RESULTS

### Systematic detection of gene losses in 62 placental mammals

To investigate whether genes associated with human disease can be inactivated in other mammals, we based our analysis on data generated by a computational gene loss detection approach that systematically screens for mutations which disrupt coding genes (18). Specifically, using a whole genome alignment between the human genome and genomes of 62 other placental mammals (23) (Supplementary Table S1), this approach detects gene inactivating mutations in non-human mammals at high accuracy, considering stop codon mutations, frameshifting insertions or deletions, mutations that disrupt splice sites and deletions of entire exons or genes (18).

Of 19 425 human genes, a total of 4317 (22.2%) genes were classified as lost in at least one of the 62 non-human mammals. We found that these genes are highly enriched in functions related to olfaction and the immune system (Table 1), which is consistent with variations in the olfactory receptor repertoire depending on species’ ecology (39) and the fast evolution of the immune system (40). Since our gene loss detection approach applies stringent filters to genome alignments and is more suited to single-copy genes (18), many members of large gene families are filtered out. Therefore, the reported olfactory receptor and immune gene enrichments may represent an underestimate. Using Dollo parsimony, which assumes that a gene loss in sister species is due to a single loss event in the ancestor of both species, we inferred on which branch(es) of the phylogenetic tree a gene loss likely happened. This showed that 2395 (55.5%) of the 4317 genes are lost repeatedly in independent lineages, suggesting that certain genes are more dispensable in evolution.

**Table 1.** Functional annotations enriched among the genes lost in at least one placental mammal

	Term ID	Number of genes	Adjusted *P*-value
**Gene Ontology: Biological Process**			
Detection of chemical stimulus involved in sensory perception	GO:0050907	390	3.5E-237
G-protein coupled receptor signaling pathway	GO:0007186	534	4.0E-99
Innate immune response	GO:0045087	228	7.4E-11
Positive regulation of peptidyl-serine phosphorylation of STAT protein	GO:0033141	16	2.8E-05
Keratinization	GO:0031424	27	3.1E-05
Peptide cross-linking	GO:0018149	28	1.5E-04
Epoxygenase P450 pathway	GO:0019373	14	1.6E-04
Drug metabolic process	GO:0017144	20	3.7E-04
Response to exogenous dsRNA	GO:0043330	23	1.4E-03
Flavonoid metabolic process	GO:0009812	16	1.2E-02
**Gene Ontology: Molecular Function**			
Olfactory receptor activity	GO:0004984	356	2.6E-225
Odorant binding	GO:0005549	81	8.0E-52
Type I interferon receptor binding	GO:0005132	16	5.0E-08
Oxidoreductase activity	GO:0016712	20	8.8E-07
Oxygen binding	GO:0019825	26	9.4E-05
Iron ion binding	GO:0005506	58	2.6E-04
Heme binding	GO:0020037	45	1.1E-02
Structural constituent of eye lens	GO:0005212	13	2.8E-02
**Human Phenotype Ontology**			
Zonular cataract	HP:0010920	18	3.1E-07
Photophobia	HP:0000613	48	3.5E-06
Posterior polar cataract	HP:0001115	28	4.6E-05
Corneal dystrophy	HP:0001131	21	4.1E-04
Progressive cataract	HP:0007834	9	1.8E-03
Dyschromatopsia	HP:0007641	24	2.6E-03
Abnormality of the macula	HP:0001103	45	2.8E-03
Complement deficiency	HP:0004431	12	3.5E-03
Progressive cone degeneration	HP:0008020	22	4.4E-03
Generalized seborrheic dermatitis	HP:0007569	6	8.9E-03
**KEGG Pathways Ontology**			
Olfactory transduction	KEGG:04740	347	2.7E-212
Drug metabolism—cytochrome P450	KEGG:00982	42	1.6E-14
Metabolism of xenobiotics by cytochrome P450	KEGG:00980	42	2.9E-13
Chemical carcinogenesis	KEGG:05204	45	3.6E-13
Autoimmune thyroid disease	KEGG:05320	29	7.7E-09
Retinol metabolism	KEGG:00830	33	2.7E-08
Systemic lupus erythematosus	KEGG:05322	51	2.5E-07
Taste transduction	KEGG:04742	37	2.9E-07
Steroid hormone biosynthesis	KEGG:00140	25	3.1E-04
Herpes simplex infection	KEGG:05168	53	4.2E-03

Enrichments were computed with gProfiler (30). Only the 10 top-ranked terms are shown.

### Lost genes are depleted in disease-associated and essential genes

**Figure 1.**
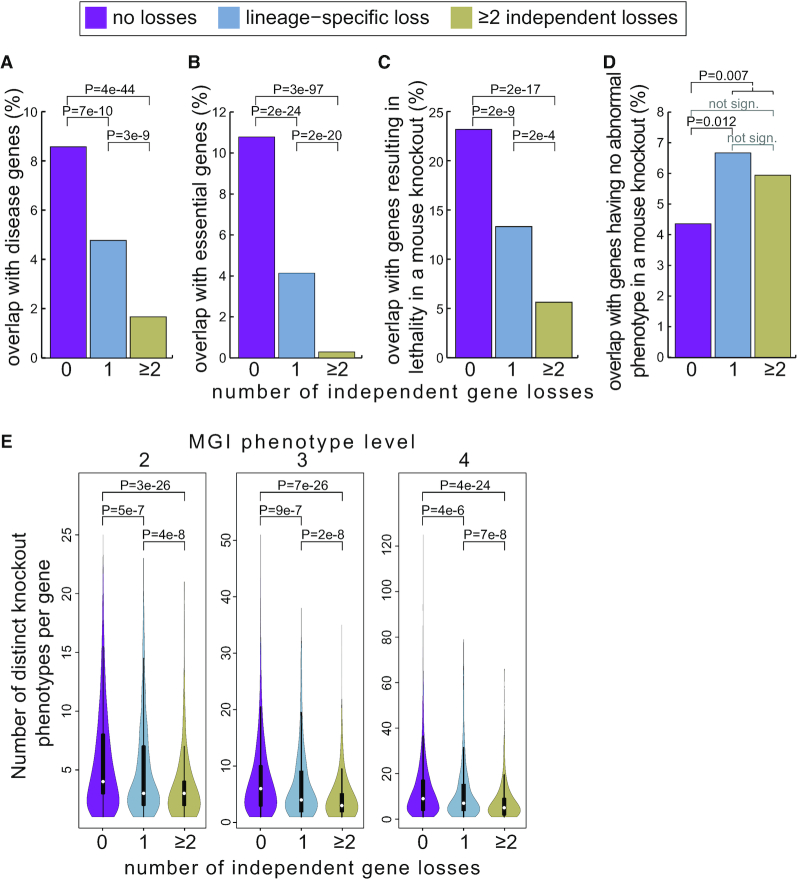
Characteristics of genes lost once or multiple times in placental mammals. Comparison of genes that are not lost in any of the 62 mammals to genes that are lost in at least one mammal. Lost genes are separated into those that are lost in a single lineage and those that are repeatedly lost in at least two independent lineages. (**A–D**) Bar charts show the percent of genes with no loss, a lineage-specific loss or multiple independent losses that (A) are implicated in human disease, (B) are essential for cellular viability, (C) result in lethality before birth in a mouse knockout and (D) have no detectable abnormal phenotype in a mouse knockout. The data show that lost genes, in particular those that are lost repeatedly, are significantly depleted in disease genes, essential genes and genes that result in lethality in a mouse knockout. In contrast, lost genes are enriched in genes that have no detectable abnormal phenotype in a mouse knockout. (E) Violin plots show that lost genes affect fewer distinct phenotypes per gene in a mouse knockout, indicating a lower degree of pleiotropy. A two-sided Fisher’s exact test was used for (A–D), a two-sided Wilcoxon rank-sum test for (E).

We tested whether lost genes are depleted in disease-associated genes by comparing lost genes to genes that are not lost in any of the 62 mammals. As expected, we found that lost genes are significantly depleted in genes implicated in human disease (Figure 1A). Consistent with this finding, lost genes are significantly depleted in genes that are essential for the viability of human cells (29) and genes that result in prenatal lethality in a mouse knockout (Figure 1B and C). In contrast, lost genes are enriched in genes that result in no detectable abnormal phenotype in a mouse knockout (Figure 1D), suggesting that these genes are more dispensable than others. Finally, since pleiotropic genes are an important contributor to human disease (41), we used the number of distinct phenotypes observed in a mouse knockout as a proxy for the degree of pleiotropy of a gene. We found that lost genes have a significantly lower degree of pleiotropy (Figure 1E). Interestingly, these characteristic properties of lost genes are further enhanced for genes that are lost independently in more than one lineage (Figure 1). Collectively, this shows that genes that are lost in mammalian evolution, and in particular those that are lost independently, are highly depleted in disease-associated genes and genes performing essential functions.

### Loss of disease genes where disease phenotypes manifest in gene-loss species

Despite the depletion of disease-associated genes, our dataset contained a number of disease-associated genes that are lost in non-human mammals. Most prominent are losses of genes implicated in eye diseases. For example, *ABCA4, BEST1, CRYBA1, EYS, GJA8, GNAT2, PDE6C, ROM1* and *SLC24A1* are implicated in disorders such as cataracts, retinitis pigmentosa, color or night blindness, or macular degeneration. These eye-related genes are mainly lost in subterranean mammals that have degenerated eyes and poor vision and their losses have been previously described (42–48). Corroborating these results, we found that lost genes are even statistically enriched in genes that function as structural lens components and genes implicated in cataracts, macular degeneration and progressive cone degeneration (Table 1).

Our dataset also contained additional disease-associated genes, where the anatomical structures affected by the human disease are degenerated or altered in gene-loss species. For example, several amelogenesis imperfecta-associated genes (*ENAM, MMP20, ACP4, AMTN*) are lost in mammals that lack tooth enamel or teeth altogether (18,49–51). Several genes associated with skin-related disorders such as peeling skin syndrome (*TGM5*), hypotrichosis (loss or reduction of hair, *DSG4*), ichthyosis (thickened and scaly skin, *ALOXE3*) or psoriasis (*KLK8*) are lost in aquatic mammals that exhibit a much thicker epidermis, a high shedding rate of epidermal cells and hair loss (18,52). *INSL3* and *RXFP4*, two genes encoding a ligand–receptor pair implicated in cryptorchidism (absence of testes from the scrotum due to their failure to descend), are lost in several Afrotheria that have naturally lost testicular descent (53). Finally, *ACOX2*, a gene whose loss-of-function mutations cause a congenital bile acid synthesis defect in humans, is lost in manatees that lack the ability to synthesize bile acids (54). In summary, non-human mammals that lost these disease genes exhibit phenotypes that resemble the human disease symptoms; however, these phenotypes do not appear to be deleterious for these mammals.

### Loss of genes where disease phenotypes do not manifest in gene-loss species

In addition to genes where disease-resembling phenotypes manifest in the gene-loss species, it may be possible to lose a disease-associated gene in the course of evolution without expressing deleterious phenotypes. A prime example is *DDB2*, a gene required for repair of UV light-induced DNA damage (55,56). Loss-of-function mutations in human *DDB2* cause xeroderma pigmentosum (57), a disease characterized by hypersensitivity to sunlight and a high risk for skin cancer (57). Similar symptoms were observed in *DDB2* knockout mice (58,59). Given these severe phenotypes, one would not expect that *DDB2* can be inactivated in other mammals. Nevertheless, we previously uncovered that *DDB2* is convergently lost in armadillos and pangolins (18), two mammals that possess thick epidermal scales. Hence, a possible explanation for the loss of *DDB2* in armadillos and pangolins is that epidermal scales protect the sun-exposed dorsal skin sufficiently well from UV light-induced DNA damage. Thus, epidermal scales may have permitted *DDB2* loss in both scaly mammals without deleterious consequences. Another example is *ABCB4*, a hepatic phospholipid transporter, whose loss in human patients results in bile canaliculi damage and severe liver disease (60). The natural loss of *ABCB4* in guinea pigs and horses is likely permissible because these two species produce less hydrophobic bile acids (61).

**Figure 2.**
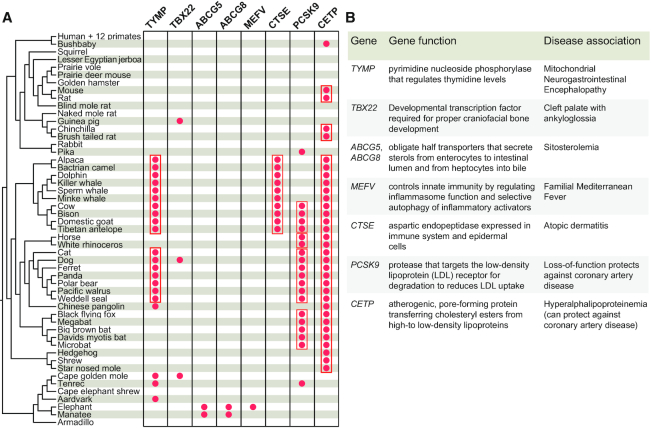
Losses of genes implicated in human disease in non-human mammals. (**A**) Overview of losses of human disease-associated genes. Red dots highlight mammals that have lost these genes. Gene losses that likely occurred in the ancestor of related species, as inferred from shared inactivating mutations, are grouped by red boxes. (**B**) Summary of gene function and the associated disease. While loss-of-function mutations in *PCSK9* and *CETP* protect humans from atherosclerosis, loss of the other five genes is associated with deleterious disease symptoms.

**Figure 3.**
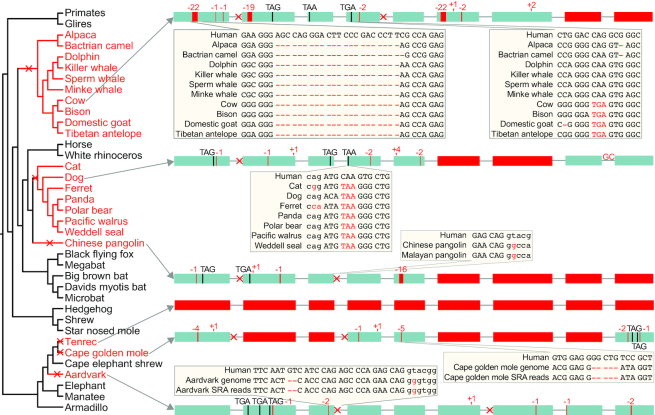
Loss of the human disease gene *TYMP* in six mammalian lineages. The left side shows the phylogeny of placental mammals with *TYMP*-loss species highlighted in red font. Based on the presence of shared inactivating mutations, this gene was inactivated at least six times in mammalian evolution, as indicated by red crosses in the tree. The right side shows the nine coding exons (boxes) of *TYMP* and all inactivating mutations of one representative species per lineage. Frameshifting insertions or deletions and stop codon mutations are indicated. A red cross near an exon indicates a splice site mutation and a filled red box represents an exon deletion. Insets illustrate that all inactivating mutations either are shared with related species or are supported by unassembled DNA sequencing reads. Smaller case letters in insets are intronic bases. While *TYMP* is completely deleted in tenrec, other lineages exhibit both exon deletions and numerous smaller inactivating mutations that are spread across the gene.

**Figure 4.**
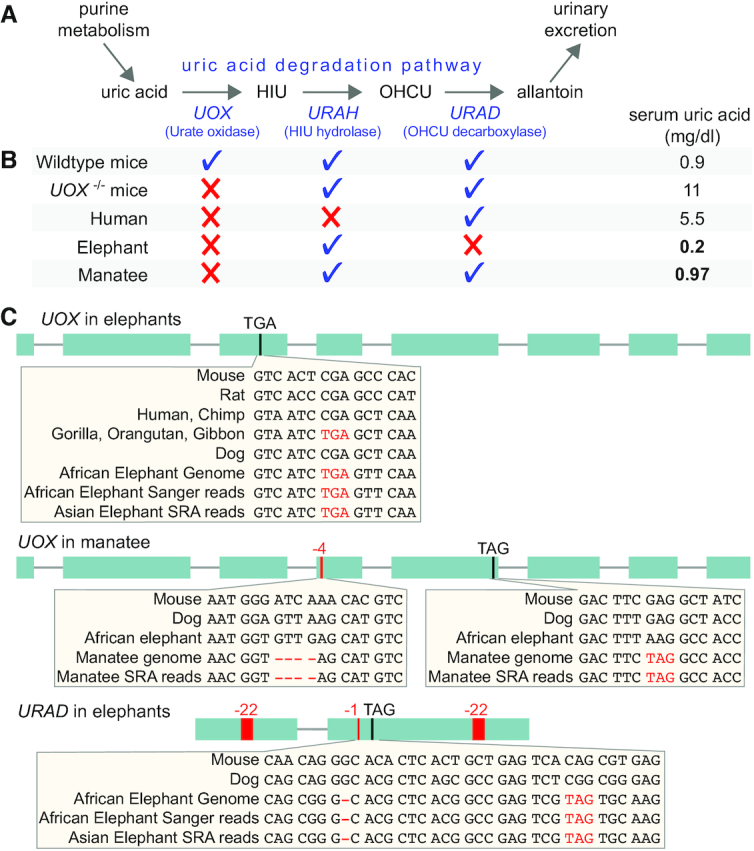
Loss of the uric acid degradation pathway in elephant and manatee. (**A**) The pathway that degrades uric acid (end product of purine metabolism) to allantoin consists of three enzymes (36): urate (the salt of uric acid at physiological pH) is oxidized by UOX to HIU (5-hydroxyisourate); URAH converts HIU to OHCU (2-oxo-4-hydroxy-4-carboxy-5-ureidoimidazoline); and OHCU is converted by URAD to allantoin, which is excreted in urine. (**B**) Loss of this pathway and a comparison of uric acid levels. A checkmark indicates an intact reading frame, a red cross indicates the presence of gene-inactivating mutations in the respective gene. Both African and Asian elephants (*Elephas Maximus*) have very low serum uric acid levels of 0.2 and 0.22 mg/dl, respectively (110,111). West Indian manatees (*Trichechus manatus*) have serum uric acid levels of 0.97 mg/dl for adult and 0.71 mg/dl for calves (112), which is similar to 1.1 mg/dl observed in the related Amazonian manatee (*Trichechus inunguis*) (113). Thus, while the loss of this pathway in human and knockout mice is associated with high serum uric acid levels, elephants and manatees have levels that are lower or comparable to the level observed in wild-type mice. (**C**) Gene inactivating mutations in coding exons of *UOX* and *URAD* in elephant or manatee. Insets illustrate that all inactivating mutations are support by unassembled DNA sequencing reads from the African elephant and the manatee. Several mutations are shared between the African and Asian elephant. The TGA stop codon mutation in elephant *UOX* exon 3 occurs at the same position as a premature stop codon in some hominoids.

Motivated by these examples, we searched for additional genes where disease-associated phenotypes are likely not present in the gene-loss species. We found six such genes (*TYMP, TBX22, ABCG5, ABCG8, MEFV, CTSE*) that are often even convergently lost in various mammals (Figures 2 and 3). Despite the fact that loss-of-function mutations in their human orthologs are implicated in rather severe disorders, disease phenotypes do not appear to manifest in these ‘natural knockout’ species, as discussed below. In addition, our search uncovered losses of the disease-relevant genes *UOX, PCSK9* and *CETP* in non-human mammals (Figures 2 and 4).

Since these gene losses are unexpected, we performed additional analyses to verify for each gene-loss species that the loss is real (Supplementary Figures S1–S9). First, we ruled out that a functional duplicated copy of these genes exist in the genome of the mammals that lost these genes. Second, we verified that the remnants of the lost genes are located in a genomic region with conserved gene order. Third, we validated the authenticity of the gene-inactivating mutations by two approaches. For those species, where genomes of closely related sister species are not yet available, we used unassembled DNA sequencing reads to confirm that inactivating mutations are supported by several reads, while there is no support for the ancestral, non-inactivating allele (Figure 3 illustrates this for *TYMP*). For those species, where genomes of closely related sister species have now become available, we confirmed that inactivating mutations are shared with sister species (Figure 3). The presence of shared inactivating mutations in independently sequenced and assembled genomes not only rules out sequencing or assembly errors, but also indicates that these mutations arose before the split of these species. The remaining non-shared and thus species-specific mutations were validated by DNA sequencing reads. Together, these analyses established that all nine genes are truly lost in the respective mammals, as summarized in Figure 2. The function and disease association of these genes and whether the evolutionary scenarios permitting gene loss are already known is described in the following.

### Loss of *TYMP* and mitochondrial neurogastrointestinal encephalopathy

We found that *TYMP* is lost in six independent lineages, comprising 21 mammals in our dataset (Figure 3 and Supplementary Figure S1). *TYMP* encodes a pyrimidine nucleoside phosphorylase that regulates thymidine levels. Mutations in human *TYMP* are associated with mitochondrial neurogastrointestinal encephalopathy (MNGIE) (62–64), a disease characterized by mitochondrial DNA alterations leading to mitochondrial dysfunction. MNGIE involves a variety of severe symptoms affecting the nervous and muscular system. The fact that *TYMP* mutations have deleterious effects in human but not in six other mammalian lineages may be explained by differences in substrate specificity between the two mammalian pyrimidine nucleoside phosphorylases, *TYMP* and *UPP1*, encoding the thymidine and uridine phosphorylase, respectively. While human UPP1 cleaves uridine but not thymidine, mouse UPP1 is known to cleave both uridine and thymidine (65,66), which explains why thymidine phosphorylase activity was still observed in a mouse *TYMP* knockout (67). Thus, multiple losses of the human disease gene *TYMP* were likely permitted in other mammals because the broader substrate specificity of the related enzyme UPP1 compensates for the lack of TYMP.

### Loss of *TBX22* and cleft palate with ankyloglossia

*TBX22* encodes a developmental transcriptional factor. We found that *TBX22* is lost in three species comprising dog, guinea pig and cape golden mole (Supplementary Figure S2). In dog and guinea pig, *TBX22* is completely deleted, which is confirmed by shared deletions in sister species (Supplementary Figure S3). Loss-of-function mutations in human *TBX22* cause a submucous cleft palate, a common birth defect that involves improper insertion of palatine muscle onto the hard palate and ankyloglossia (tongue-tie) (68,69). These phenotypes match the expression of *TBX22* in the palatal shelves and the base of the tongue (70) and support the function of *TBX22* as a key factor for palatine bone development (71). *TBX22* knockout mice also show a submucous cleft palate and ankyloglossia; thus they resemble the phenotype observed in humans with *TBX22* mutations (71). In contrast to human or mouse, guinea pigs and dogs are not known to naturally have a cleft palate (72). Golden moles also have a large and well-developed palatine without observed cleft palates (73). Thus, these three independent mammals do not appear to rely on *TBX22* for proper craniofacial development anymore. A possible explanation is that redundancy with other T-box transcription factors that are expressed in developing craniofacial tissues (74,75) led to a rewiring of the underlying regulatory network in these species, which may have permitted loss of *TBX22*. Interestingly, a cleft palate is spontaneously observed in certain dog breeds and has been linked to mutations in the *ADAMTS20* locus (72). This raises the possibility that *TBX22* loss in dogs makes them more susceptible to developing a cleft palate if other genes are mutated.

### Loss of *ABCG5* and *ABCG8* and sitosterolemia

We found that elephants and manatees have independently lost the *ABCG5* and *ABCG8* genes (Supplementary Figures S4 and S5). Several gene-inactivating mutations are shared between the African and Asian elephant, indicating that *ABCG5* and *ABCG8* were already lost in the ancestor of both elephant species. These two genes encode the half-transporters sterolin-1 and sterolin-2. Sterolins have a dual role in sterol excretion, and are expressed at the brush border membrane of enterocytes and the canalicular membrane of hepatocytes (76,77). In enterocytes, sterolins transport passively absorbed sterols back into the intestinal lumen. In hepatocytes, they secrete sterols into the bile. Since sterolins transport phytosterols (plant sterols) at a much higher rate than cholesterol, their activity results in a plasma phytosterol level that is substantially lower than the cholesterol level. Mutations in human *ABCG5* or *ABCG8* cause sitosterolemia, a disease characterized by increased absorption and decreased biliary excretion of dietary phytosterols such as the common plant sterol β-sitosterol (78). The resulting increased plasma sterol levels in turn cause xanthomas (deposition of sterol-rich material) on tendons and joints, atherosclerosis, and coronary artery disease. Similarly, a double knockout of *ABCG5* and *ABCG8* in mouse results in substantially increased plasma phytosterol levels (79). Given that loss of *ABCG5* and *ABCG8* reduces cholesterol synthesis in both human and mouse (79,80), it is possible that the loss of these genes could be beneficial for strictly herbivorous elephants and manatees by saving energy necessary to synthesize cholesterol, which occurs only in small amounts in an herbivorous diet. However, the loss of *ABCG5* and *ABCG8* raises the question of whether sitosterol accumulates in these species, which is not known to the best of our knowledge. Thus, it would be interesting to investigate how elephants and manatees manage sitosterol levels or alternatively why high levels of sitosterol have no deleterious effects.

### Loss of *MEFV* and familial Mediterranean fever

The African elephant lost *MEFV* (Supplementary Figure S6), a gene that is linked to the autoinflammatory disease familial Mediterranean fever (FMF) (81,82). As for *ACBG5/8*, we found that several gene-inactivating mutations are shared with the Asian elephant, indicating that *MEFV* was already lost in the ancestor of both elephants. FMF is characterized by recurrent fever attacks, inflammation of the serosa tissues and other symptoms (83). *MEFV* encodes the protein pyrin that controls innate immunity by regulating inflammasome function and selective autophagy of inflammatory activators (84,85). Macrophages from *MEFV* knockout mice show increased interleukin (IL) 1β release after stimulating inflammasome assembly, establishing *MEFV* as an inhibitor of IL-1β release (86). This is consistent with anti IL-1 drugs being an effective treatment for some FMF patients (87). Given that FMF symptoms are not known to manifest in elephants, this species likely evolved a different way to control the innate immune response and research on elephant immune system cells could shed new light on FMF.

### Loss of *CTSE* and atopic dermatitis

We found that mammals belonging to the cetartiodactyla clade (alpacas, cetaceans, cow, goat and others) have lost the *CTSE* gene (Supplementary Figure S7), which encodes the aspartic proteinase cathepsin E that is expressed in cells of the immune system and the epidermis. Cathepsin E plays a role in macrophage autophagy and in the terminal differentiation of keratinocytes (88,89). A mouse knockout results in itching, encrusted and erythematic skin lesions that resemble the symptoms observed in the common inflammatory skin disease atopic dermatitis (90). *CTSE* knockout mice exhibit a reduced turnover of interleukins that accumulate systemically, which likely initiates the development of atopic dermatitis (90). Consistent with an involvement of *CTSE* in this disease, human atopic dermatitis patients show reduced *CTSE* expression (90). While several genes associated with skin disorders are specifically lost in cetaceans (18,52), *CTSE* is special since it is lost not only in fully aquatic cetaceans but also in terrestrial mammals. Since symptoms of atopic dermatitis are not known to occur in cetartiodactyla, it remains to be studied which mechanisms permitted the loss of *CTSE* in these species.

### Loss of the uric acid degradation pathway is not unique to hominoids

It is known that humans and other hominoids (chimp, gorilla, orangutan, gibbon) have lost the *UOX* (urate oxidase) gene (35), a key enzyme in the pathway that degrades uric acid (the end product of purine metabolism) to allantoin for urinary excretion (Figure 4A). The loss of this pathway contributed to an increased level of serum uric acid in humans (5.5 versus 0.5–1 mg/dl in other mammals, Figure 4B) (91,92). Consistent with this, *UOX* knockout in mouse increases the serum uric acid level from 0.9 mg/dl in wild-type mice to 11 mg/dl in knockout mice (38). Several hypotheses suggest that high uric acid levels may have been beneficial during hominoid evolution. Since uric acid serves as a powerful antioxidant, higher levels may be linked to cancer resistance and increased longevity (92,93). In addition, high uric acid levels can maintain blood pressure on a low salt diet that was prevalent in hominoids during the Miocene (92,94). However, sustained elevated uric acid levels come at the cost of a high risk for gout, an inflammatory arthritis disease caused by accumulation of uric acid crystals in joints.

Strikingly, we discovered that, like hominoids, elephants and manatees have inactivating mutations in *UOX* and thus convergently lost the uric acid degradation pathway (Figure 4C). In contrast to humans or *UOX* knockout mice, both elephants and manatees have low serum uric acid levels (<1 mg/dl; Figure 4B), raising the question how both species manage to achieve such low levels despite lacking a functional uric acid degradation pathway. In humans, purine-rich food like meat or seafood is an additional risk factor for hyperuricemia and gout; however, different diets have generally only a small effect on serum uric acid levels and vegans have the highest uric acid levels (95). As strict herbivores, elephants and manatees might avoid purine-rich food. However, diet alone does not provide a full explanation for the loss of the uric acid degradation pathway in these two lineages, as many other herbivores maintain this pathway. Investigating how elephants and manatees metabolize uric acid may provide new insights into uric acid homeostasis and how to achieve low serum levels despite the loss of the uric acid degradation pathway.

### Gene losses that may protect from coronary artery disease

While inactivation of the above-mentioned disease genes has deleterious effects for humans, loss-of-function mutations in genes can also be associated with a reduced risk for disease. Two prominent examples are *PCSK9* and *CETP*, where loss-of-function mutations are thought to protect from coronary artery disease (96–98). PCSK9 reduces the hepatic uptake of atherosclerosis-promoting low-density lipoproteins (LDL) by targeting the LDL receptor for degradation (99). While *PCSK9* gain-of-function mutations cause hypercholesterolemia (100,101), loss-of-function mutations in the human or mouse gene result in low LDL cholesterol levels (96,102–104). CETP transfers cholesteryl ester from high-density lipoproteins (HDCs) to LDLs in exchange for triglycerides (105). Mutations in human CETP are associated with increased HDL cholesterol levels and reduced levels of the atherosclerosis-promoting LDL cholesterol (97,106). We found that these two genes have been inactivated many times in non-human mammals: *PCSK9* is lost in at least six independent lineages totaling 20 mammals and *CETP* is lost in at least four independent lineages comprising a total of 33 mammals in our dataset (Figure 2A; Supplementary Figures S8 and S9). Interestingly, many mammals have lost both *PCSK9* and *CETP*.

## DISCUSSION

By analyzing gene loss data of 62 placental mammals, we showed that lost genes are characterized by a lower degree of pleiotropy and a depletion in essential and diseases-associated genes. Nevertheless, there are a number of disease-associated genes that are truly lost in non-human mammals. These genes largely fall into three classes. First, in most of these cases, the symptoms that characterize the human disease do resemble natural traits of mammals that lost these genes. Prominent examples include losses of genes associated with human eye-, skin- or teeth-related disorders in mammals that exhibit degenerated eyes, skin alterations or loss of tooth enamel. Importantly, these altered traits are not deleterious or may even be adaptive in the environment and ecological niche of these mammals.

Second, we discovered losses of disease-associated genes in non-human mammals where disease-associated phenotypes do not appear to manifest. These rather unexpected findings suggest that other genes or alternative mechanisms in these mammals may be able to substitute for the function of the disease-associated gene, thus rendering the gene redundant and permitting its loss in these species. For example, non-human mammals likely possess a UPP1 enzyme with a broader substrate specificity, which may have permitted the loss of the mitochondrial neurogastrointestinal encephalopathy-causing gene *TYMP* in six mammalian lineages by making it functionally redundant. The loss of the cleft palate associated transcription factor *TBX22* in three mammalian lineages that do not exhibit such a craniofacial defect may have been permissible by changes in the developmental gene regulatory network that led other transcription factors to assume the role of *TBX22*. Similar, yet unknown, mechanisms could explain the lack of disease-associated phenotypes in non-human mammals that lost genes implicated in lipid metabolism (*ABCG5, ABCG8*), uric acid metabolism (*UOX*) and immune-related human diseases (*MEFV, CTSE*). Strikingly, most of these genes are even convergently lost in different mammalian lineages (Figure 2A). Investigating which genes or mechanisms allow these species to be ‘natural knockouts’ for disease-implicated genes without exhibiting disease symptoms would be an interesting future direction.

Third, loss-of-function mutations in genes may sometimes be protective against disease. For example, a stop codon mutation in *CASP12* in certain human populations was likely selected for a decreased risk for sepsis (107) and a frameshifting deletion in the chemokine receptor gene *CCR5* protects humans from HIV infection (108). Here, we show that *PCSK9* and *CETP*, two genes whose inactivation is thought to protect humans against coronary artery disease, are lost in many independent mammalian lineages. The widespread losses of *PCSK9* and *CETP* might be an indication that inactivating these genes is also advantageous for non-human mammals, which would add to recent studies providing evidence that losing ancestral coding genes in the course of evolution can be beneficial under special circumstances (18,24,109). Whether losing *PCSK9* or *CETP* is beneficial for non-human mammals and what the potential benefit is remains to be investigated.

The rapidly growing number of sequenced genomes will make it possible to extend systematic screens for inactivated genes to many other species. This will certainly uncover additional species that are natural knockouts for human disease-implicated genes and will eventually reveal which genes are truly essential.

## DATA AVAILABILITY

All analyzed genome assemblies (Supplementary Table S1) are publicly available on the UCSC genome browser and from NCBI. Sequencing read data (Supplementary Table S1) is publicly available from the NCBI Trace and Sequence Read Archive. Source code is available at https://github.com/hillerlab/GeneLossPipe. All validated gene inactivating mutations are shown in the Supplementary Figures S1–S9.

## Supplementary Material

lqz012_Supplemental_Files

## Notes

Present address: Virag Sharma, CRTD-DFG Center for Regenerative Therapies Dresden, Carl Gustav Carus Faculty of Medicine, Technische Universität Dresden, 01307 Dresden; Paul Langerhans Institute Dresden (PLID) of the Helmholtz Center Munich at University Hospital Carl Gustav Carus and Faculty of Medicine, Technische Universität Dresden, 01307 Dresden; German Center for Diabetes Research (DZD), 85764, Munich, Neuherberg, Germany.
