# Supplementary material for: Losses of human disease-associated genes in placental mammals
Source: NAR Genom Bioinform. 2019 Oct 24;2(1):lqz012. doi: 10.1093/nargab/lqz012 (PMC7671337; doi:10.1093/nargab/lqz012)
Supplement: lqz012_Supplemental_Files [file lqz012_supplemental_files.zip › Supplement.pdf]

Supplementary Material for

Losses of human disease-associated genes in placental mammals

Virag Sharma[1,2,3#] and Michael Hiller[1,2,3*]

[1]Max Planck Institute of Molecular Cell Biology and Genetics, Dresden, Germany
[2]Max Planck Institute for the Physics of Complex Systems, Dresden, Germany
[3]Center for Systems Biology Dresden, Germany

*To whom correspondence should be addressed:
Michael Hiller
Computational Biology and Evolutionary Genomics, Max Planck Institute of Molecular Cell Biology and Genetics & Max Planck Institute for the Physics of Complex Systems, Dresden, Germany.
Tel: +49 351 210 2781
Fax: +49 351 210 1209
Email: hiller@mpi-cbg.de

[#] current affiliations: CRTD-DFG Center for Regenerative Therapies Dresden, Carl Gustav Carus Faculty of Medicine, Technische Universität Dresden, Dresden; Paul Langerhans Institute Dresden (PLID) of the Helmholtz Center Munich at University Hospital Carl Gustav Carus and Faculty of Medicine, Technische Universität Dresden, Dresden; German Center for Diabetes Research (DZD), Munich, Neuherberg, Germany.

The Supplementary Material contains

- Supplementary Figures 1-9
- Supplementary References.

Supplementary Table 1 is provided in a separate Excel file.

General figure legend for Supplementary Figures 1, 2 and 4-9.

The exon-intron structure of the human gene is visualized together with inactivating mutations in other non-human mammals as inferred from CESAR (1,2) alignment. Boxes are exons (proportional to their size), introns are horizontal lines. Only coding portions of exons are shown. A filled red box is an exon deletion and a filled grey box represents missing genomic sequence. A black vertical line indicates an in-frame stop codon with the corresponding triplet above. A red (blue) vertical line is a frameshifting (frame-preserving) deletion and a red (blue) arrow head is a frameshifting (frame-preserving) insertion. The size of deletions or insertions is given on top of the mutation. Frame-preserving insertions or deletions smaller than 15 bp are not shown. Splice site mutations are indicated before or after the exon and the mutated donor or acceptor dinucleotide is shown.

All smaller inactivating mutations (frameshifting insertions and deletions, stop codon and splice site mutations) that are shown in these figures are validated either by unassembled sequencing reads from the same species or by the presence of the same mutation in the independently sequenced and assembled genome of sister species. The Tibetan antelope is an exception as no unassembled sequencing reads are available, which prevents a validation of antelope-specific mutations. However, *TYMP*, *CTSE*, *PCSK9* and *CETP*, which are lost in the Tibetan antelope, all have mutations that are shared with sister species, indicating that these four genes are truly lost in the Tibetan antelope.

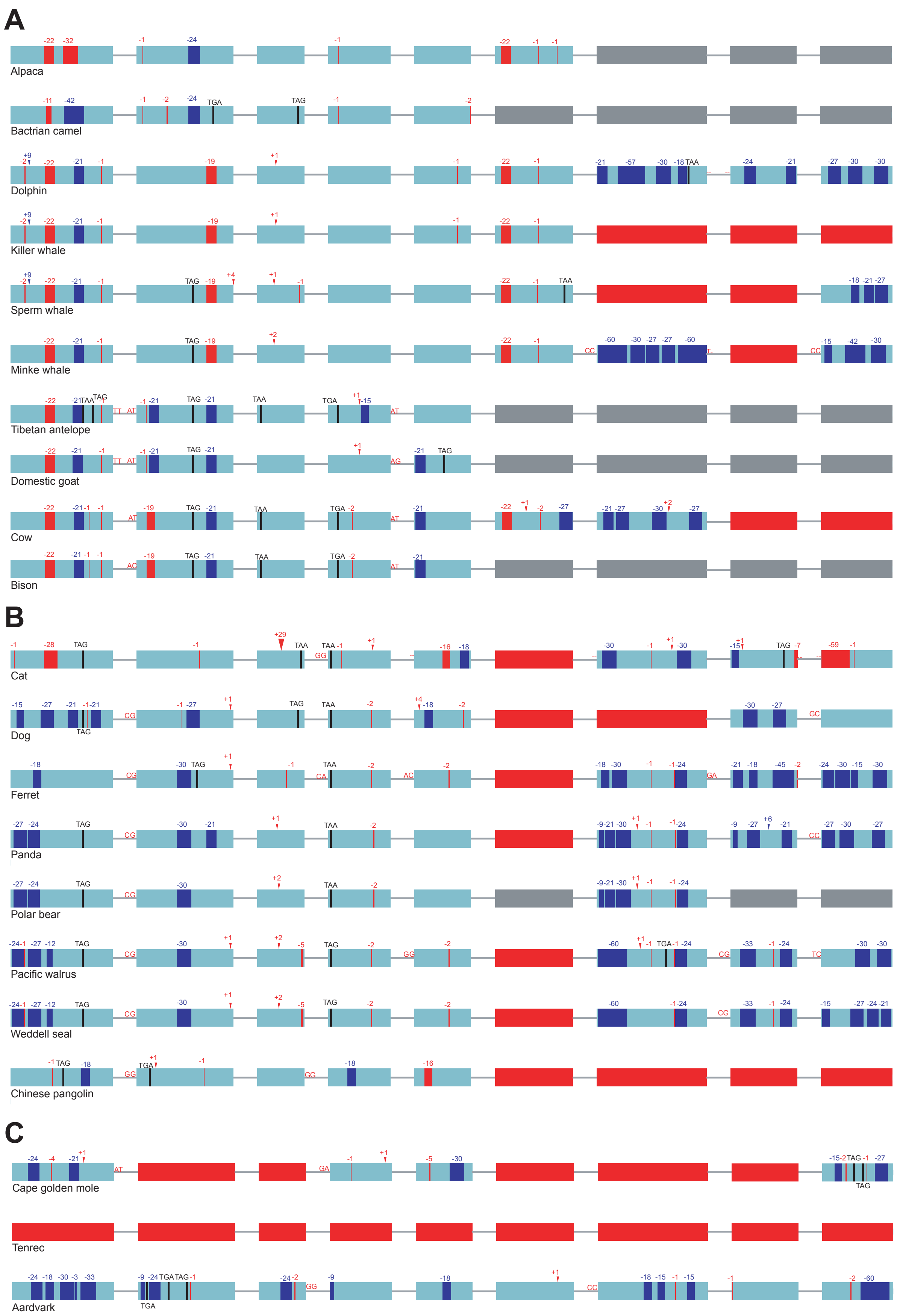

Supplementary Figure 1: Inactivating mutations in *TYMP* in Cetartiodactyla (A), Carnivora (B), and cape golden mole, tenrec and aardvark (C). See page 2 for the general legend. Shared inactivating mutations that support a common loss in

Cetartiodactyla and Carnivora are shown in Figure 3 main text. Of all the gene-loss species shown here, only the loss of *TYMP* in cow has been reported previously (3).

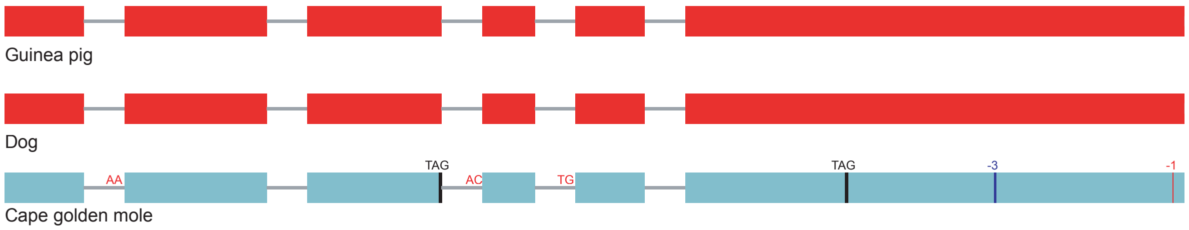

Supplementary Figure 2: Mutations in *TBX22* in guinea pig, dog and cape golden mole.
See page 2 for the general legend. Of these three species, only the loss of *TBX22* in dog has been reported previously (4).

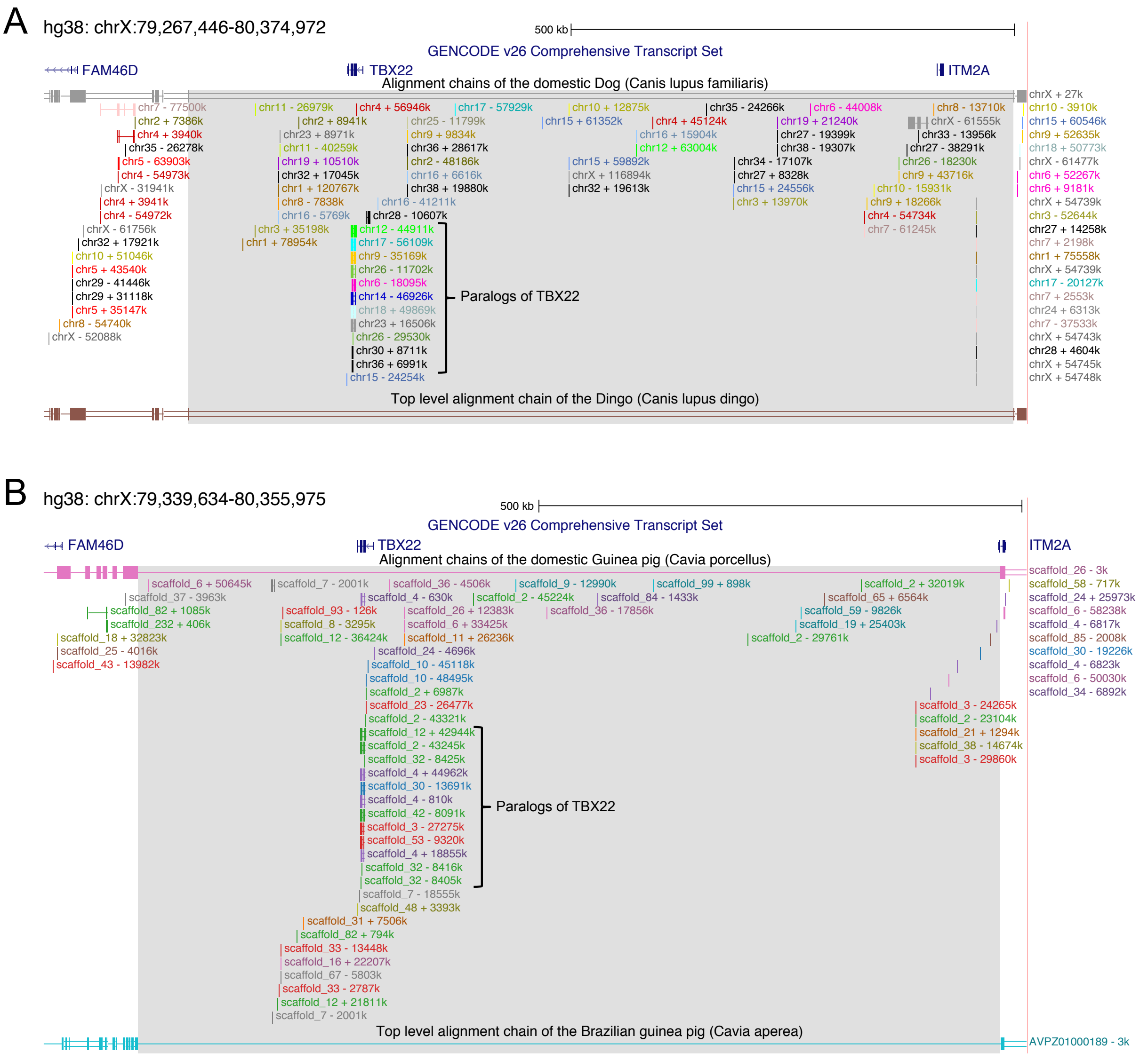

Supplementary Figure 3: Deletion of *TBX22* in dog and guinea pig.

(A) A UCSC genome browser (5) screenshot displays co-linear alignment chains between human and the domestic dog, which shows that *TBX22* is completely deleted in dog. To confirm that this deletion is not an assembly error, we aligned human to the genome of the dingo, a closely-related species in the same genus as dog. These alignment chains show that the deletion is shared between both species (grey background). Chains that align paralogs of *TBX22* such as *TBX15*, *TBX18*, *TBX4* etc. are indicated.

(B) To confirm the deletion of *TBX22* in the domestic guinea pig, we aligned the genome of the Brazilian guinea pig, which belongs to the same genus. As for dog, the deletion of *TBX22* is shared between both guinea pig species.

Aligning blocks in co-linear alignment chains are represented as boxes, single or double lines represent deletions or larger unaligning regions. For clarity, only the top alignment chain is shown for dingo and Brazilian guinea pig.

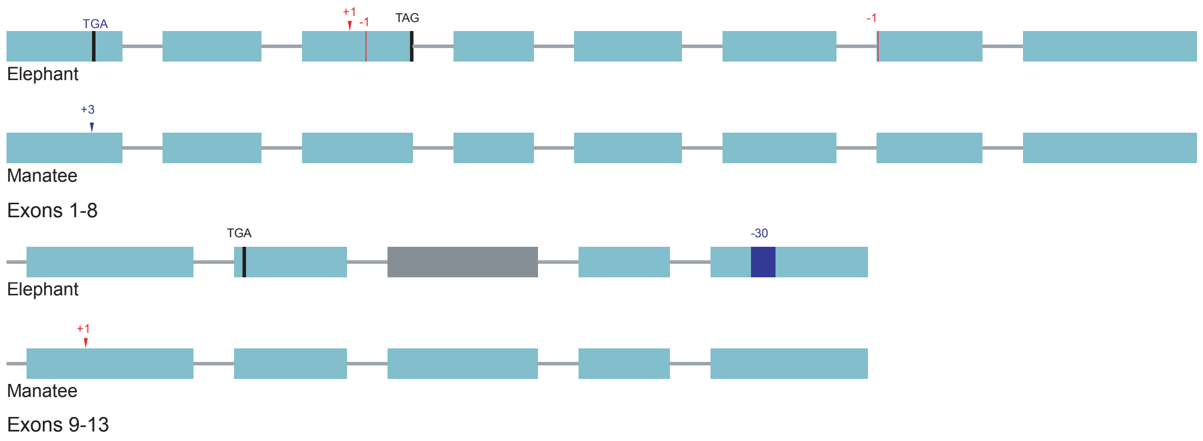

Supplementary Figure 4: Inactivating mutations in *ABCG5* in the African elephant and manatee.

See page 2 for the general legend. For the African elephant, several inactivating mutations are shared with the Asian elephant (all shown mutations are also validated by Sanger sequencing reads of the African elephant).

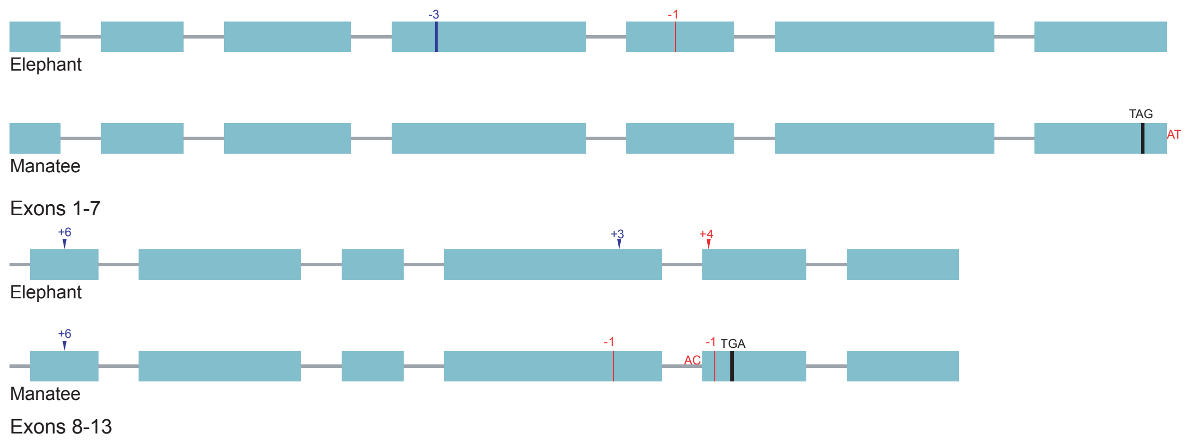

Supplementary Figure 5: Inactivating mutations in *ABCG8* in the African elephant and manatee.

See page 2 for the general legend. The 1 bp deletion in exon 5 in the African elephant is also present in sequencing reads of the Asian elephant (6), supporting that both *ABCG5* and *ABCG8* were already inactivated in the common ancestor of both elephants.

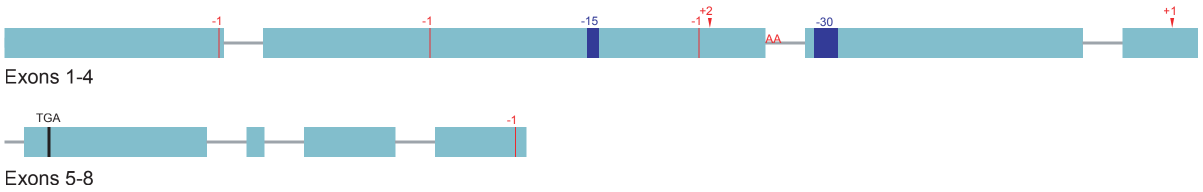

Supplementary Figure 6: Inactivating mutations in *MEFV* in the African elephant. See page 2 for the general legend. Several inactivating mutations also found in sequencing reads of the Asian elephant (6), showing that they are shared between both elephant species.

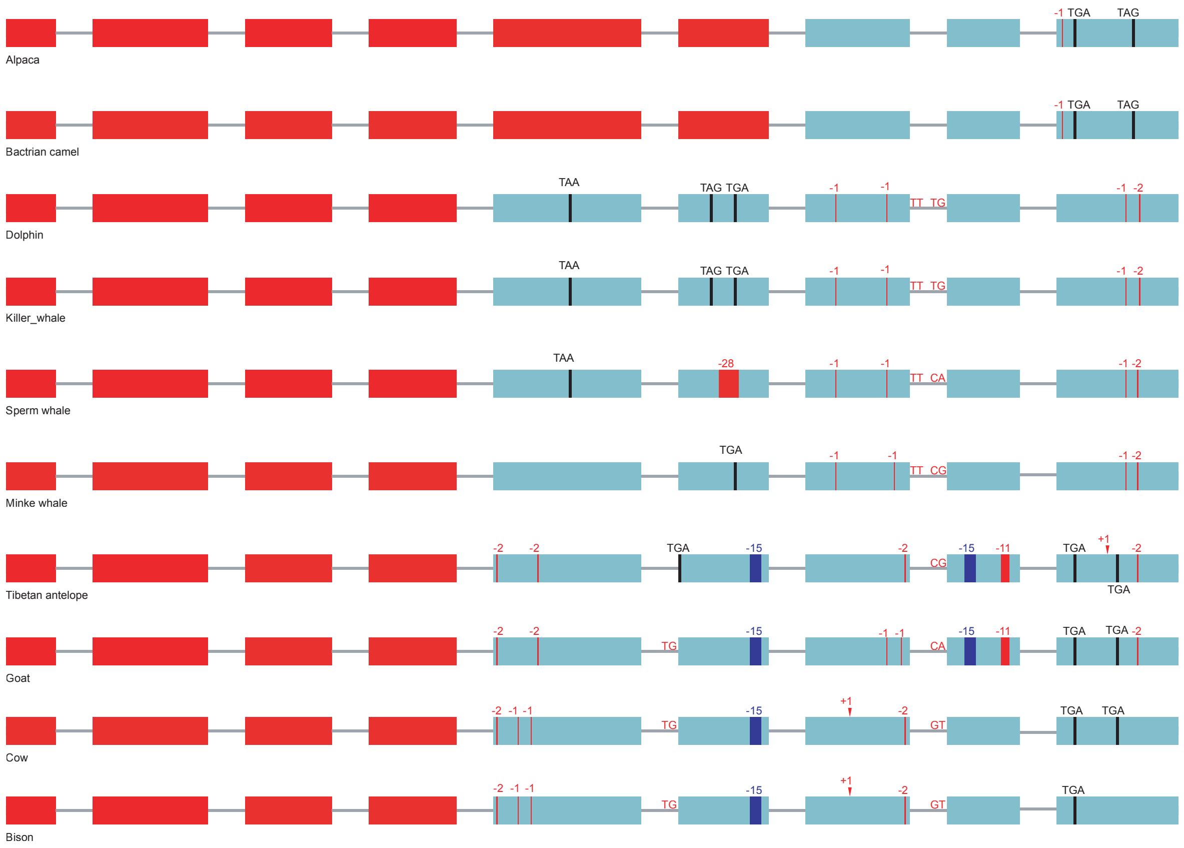

Supplementary Figure 7: Inactivating mutations in *CTSE* in Cetartiodactyla.
See page 2 for the general legend. The deletion of exons 1 to 4 is shared between Cetartiodactyla species, showing that this gene was already lost in their common ancestor.

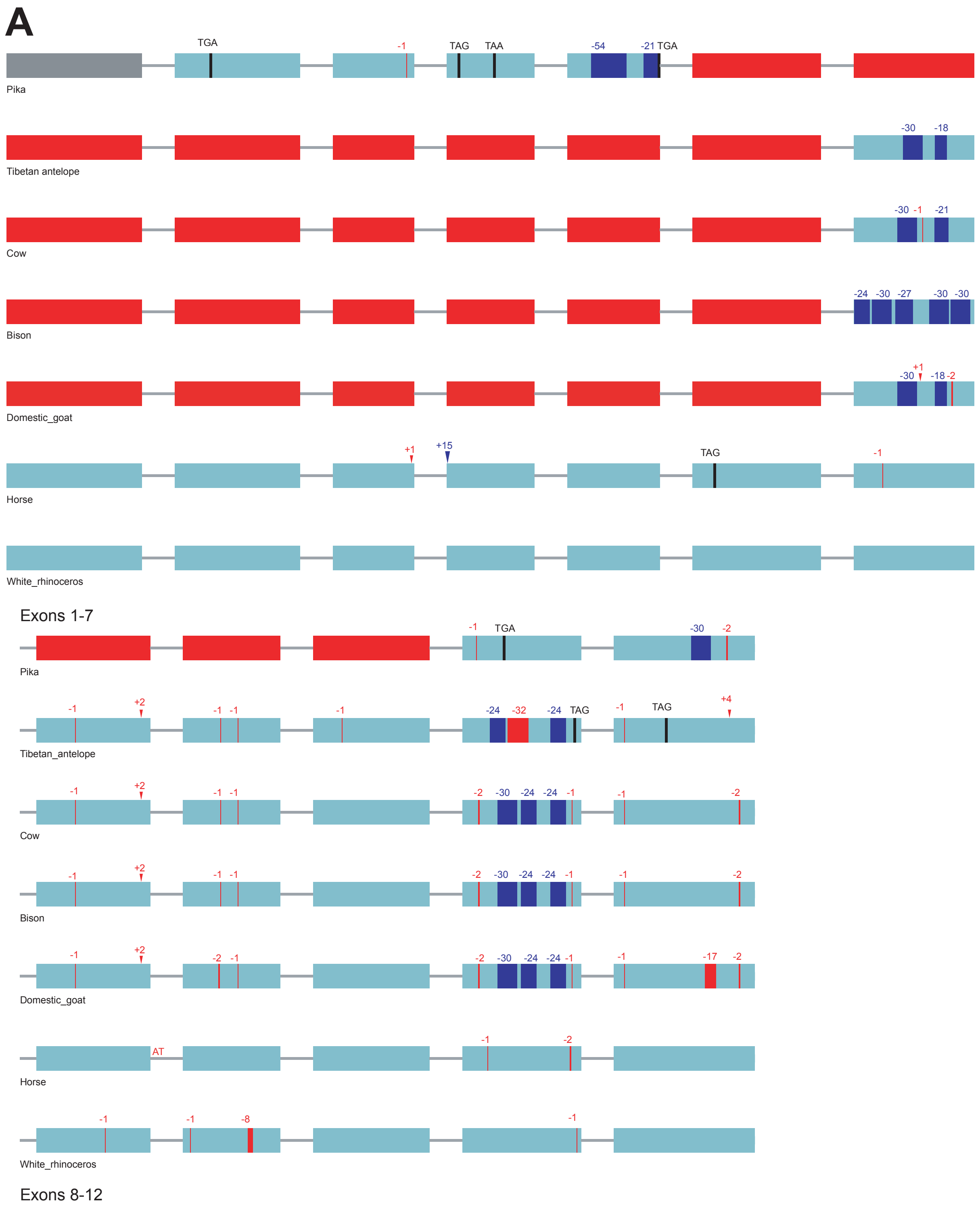

Supplementary Figure 8(A): Inactivating mutations in *PCSK9* in the pika, Ruminantia and Perissodactyla.

See page 2 for the general legend. Several mutations are shared between Ruminantia, including the deletion of exons 1 to 6. In the horse and rhinoceros, exon 11 has a 1 bp frameshifting deletion that is shared between both species; however, the rhinoceros has another 1 bp insertion upstream which is why CESAR (1,2) does not report an inactivating mutation. Of all the gene-loss species shown in panels A, B and C, only the loss of *PCSK9* in cow has been reported previously (7).

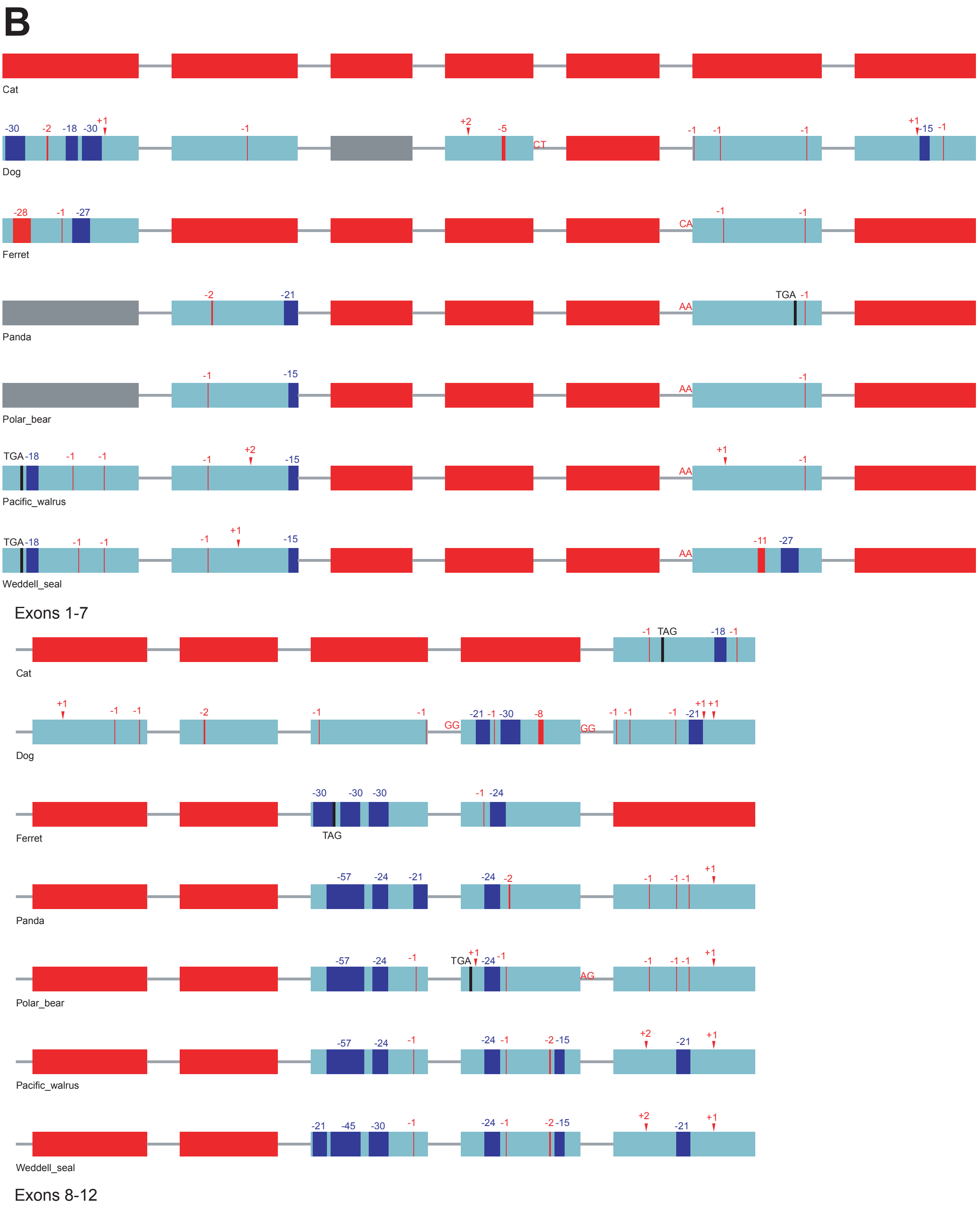

Supplementary Figure 8(B): Inactivating mutations in *PCSK9* in Carnivora.
See page 2 for the general legend. Exon 6 has a 1 bp frameshifting deletion that is shared across almost species. The respective region was presumably later deleted in the cat and Weddell seal.

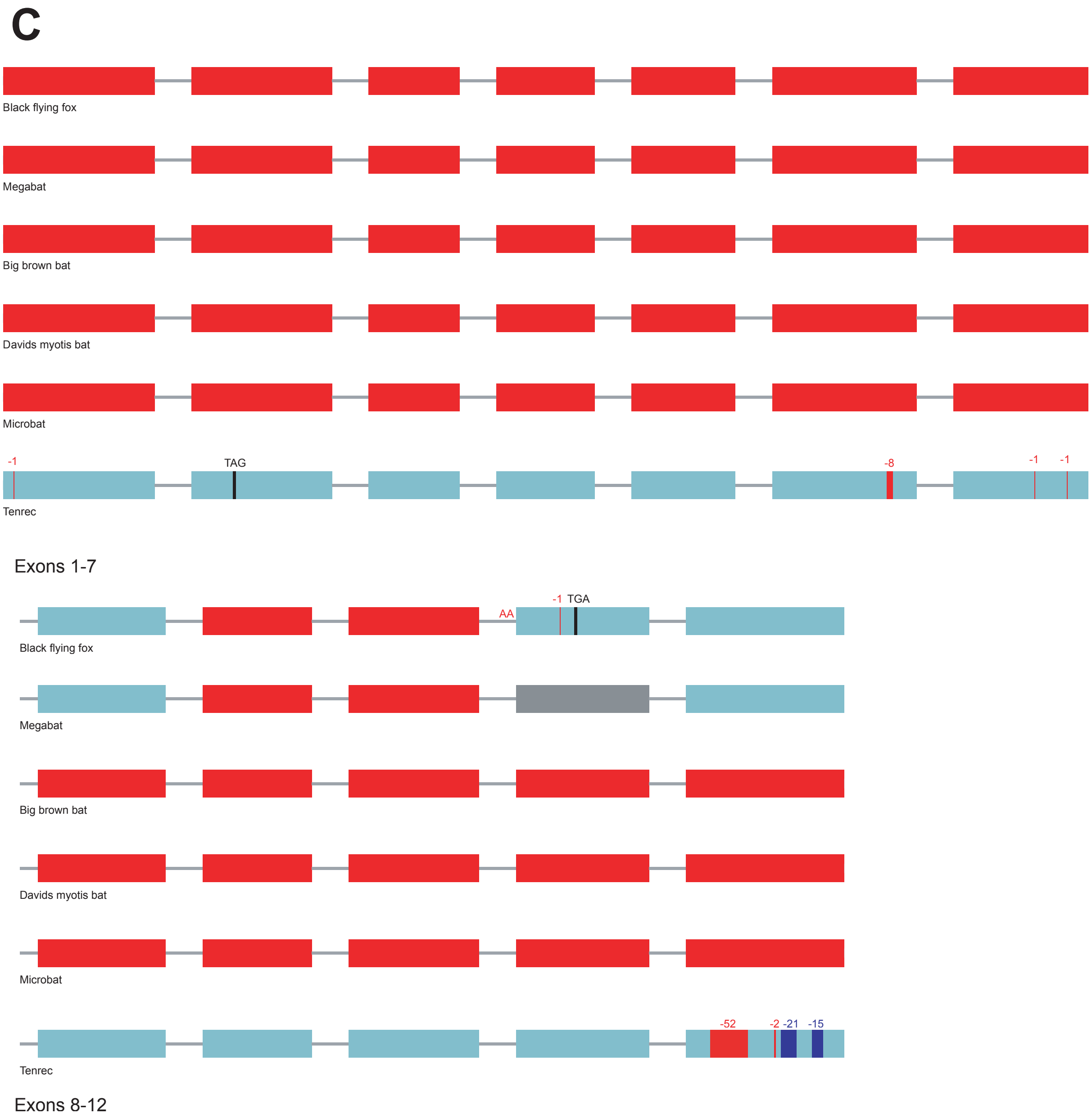

Supplementary Figure 8(C): Inactivating mutations in *PCSK9* in Chiroptera and the tenrec.

See page 2 for the general legend. In Chiroptera, large deletions overlap between species, which is why we conservatively assume a single loss of *PCSK9* in this clade; however, it is also possible that this gene was lost independently in Chiroptera.

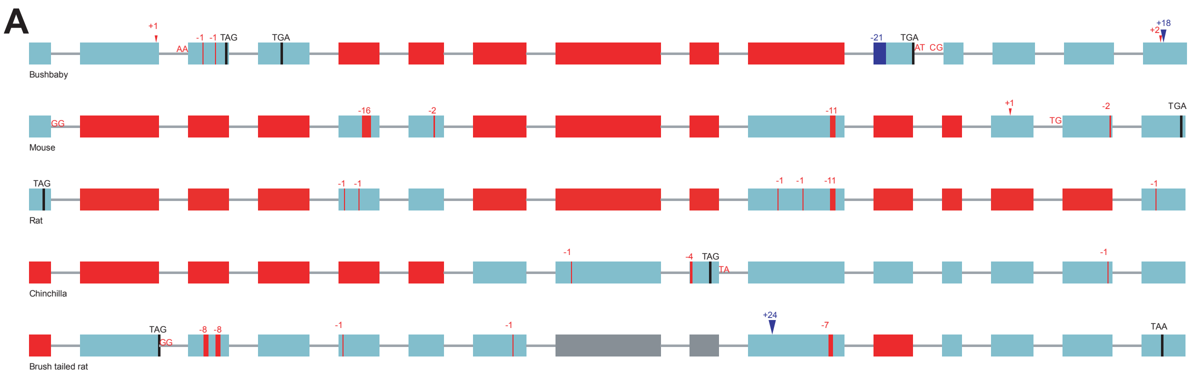

Supplementary Figure 9(A): Inactivating mutations in *CETP* in the bushbaby, mouse and rat, and chinchilla and brush-tailed rat.

See page 2 for the general legend. Mouse and rat share an 11 bp deletion in exon 10 and the deletion of exons 2 to 4. Chinchilla and brush-tailed rat likely share the deletion of exon 1, which has similar though not identical breakpoints. Of all the gene-loss species shown in panels A-D, only the loss of *CETP* in mouse and rat has been reported previously (8). However, further supporting the gene losses reported here, *CETP* loss perfectly correlates with the absence of CETP enzymatic activity in mouse, rat, dog, cat, cow, goat, horse and pig (9,10).

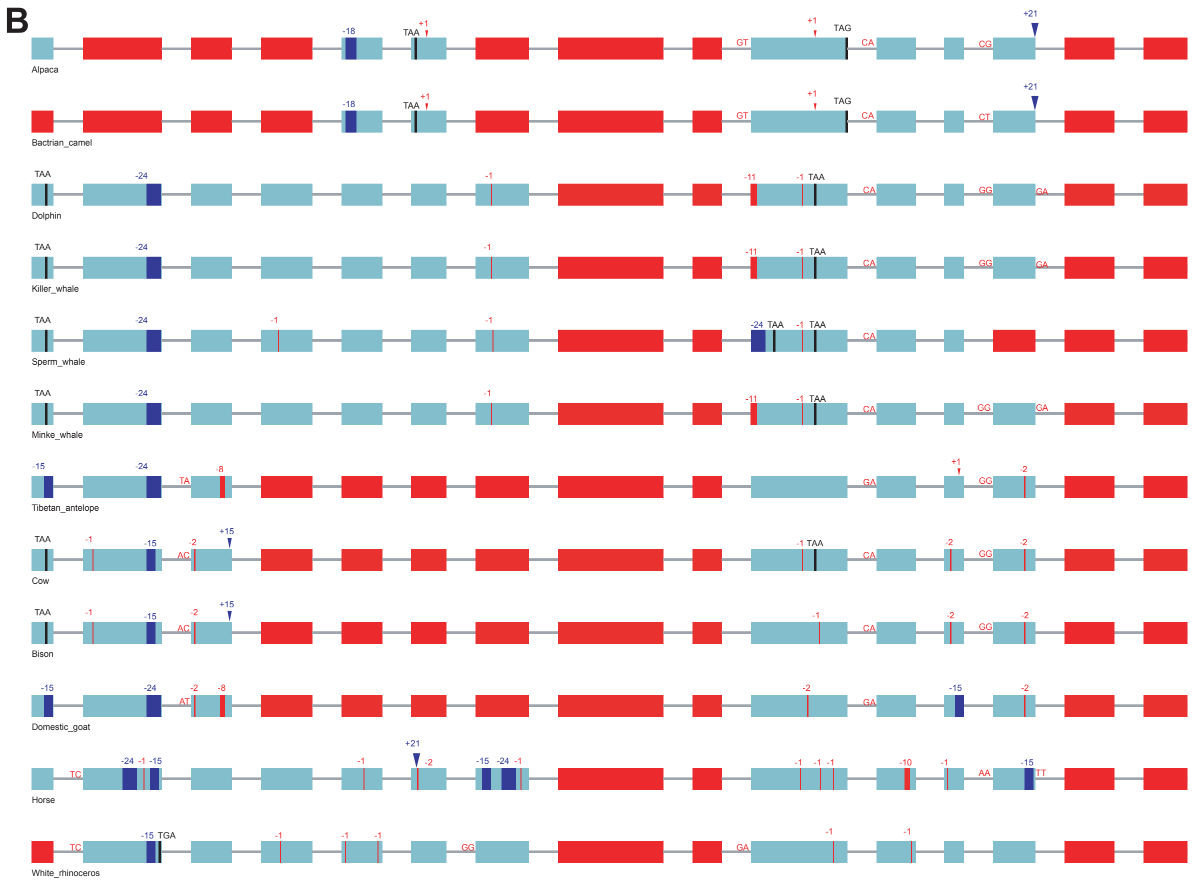

Supplementary Figure 9(B): Inactivating mutations in *CETP* in Cetartiodactyla and Perissodactyla.

See page 2 for the general legend. The deletion of exons 14 and 15 is most likely shared between all Laurasiatheria species, indicating an ancestral gene loss.

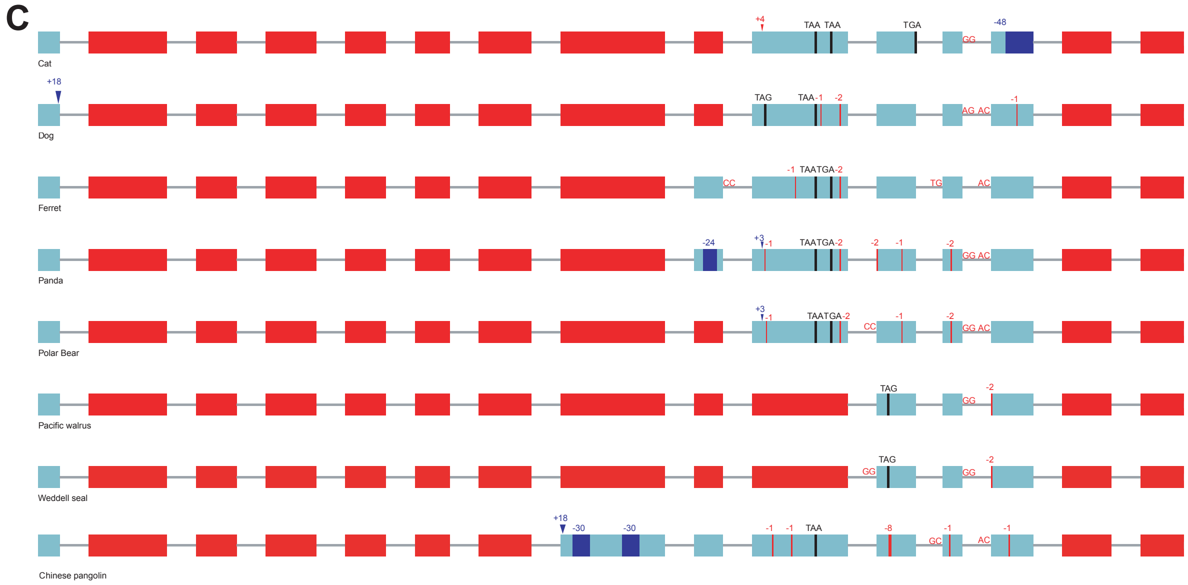

Supplementary Figure 9(C): Inactivating mutations in *CETP* in Carnivora and the pangolin. See page 2 for the general legend.

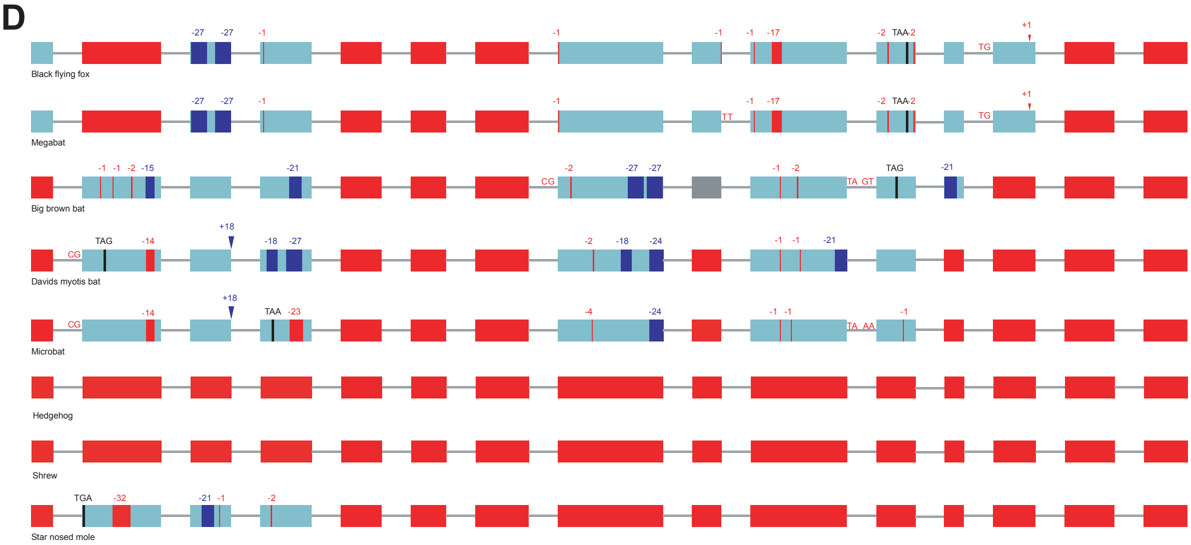

Supplementary Figure 9(D): Inactivating mutations in *CETP* in Chiroptera, hedgehog, shrew and star nosed mole. See page 2 for the general legend. The entire gene appears to be absent from the genomes of the hedgehog and shrew, and the respective unaligning regions that are flanked by up- and downstream aligning blocks are markedly shorter than in human, which is consistent with a larger deletion. However, the respective region overlaps an assembly gap in both species, therefore an upgraded genome assembly is required to confirm the loss of *CETP* in hedgehog and shrew.

Supplementary References

1. Sharma, V., Elghafari, A. and Hiller, M. (2016) Coding exon-structure aware realigner (CESAR) utilizes genome alignments for accurate comparative gene annotation. *Nucleic Acids Res*, **44**, e103.
2. Sharma, V., Schwede, P. and Hiller, M. (2017) CESAR 2.0 substantially improves speed and accuracy of comparative gene annotation. *Bioinformatics*, **33**, 3985-3987.
3. Elsik, C.G., Tellam, R.L., Worley, K.C., Gibbs, R.A., Muzny, D.M., Weinstock, G.M., Adelson, D.L., Eichler, E.E., Elnitski, L., Guigo, R. *et al.* (2009) The genome sequence of taurine cattle: a window to ruminant biology and evolution. *Science*, **324**, 522-528.
4. Derrien, T., Theze, J., Vaysse, A., Andre, C., Ostrander, E.A., Galibert, F. and Hitte, C. (2009) Revisiting the missing protein-coding gene catalog of the domestic dog. *BMC Genomics*, **10**, 62.
5. Casper, J., Zweig, A.S., Villarreal, C., Tyner, C., Speir, M.L., Rosenbloom, K.R., Raney, B.J., Lee, C.M., Lee, B.T., Karolchik, D. *et al.* (2018) The UCSC Genome Browser database: 2018 update. *Nucleic Acids Res*, **46**, D762-D769.
6. Reddy, P.C., Sinha, I., Kelkar, A., Habib, F., Pradhan, S.J., Sukumar, R. and Galande, S. (2015) Comparative sequence analyses of genome and transcriptome reveal novel transcripts and variants in the Asian elephant Elephas maximus. *Journal of biosciences*, **40**, 891-907.
7. Cameron, J., Holla, O.L., Berge, K.E., Kulseth, M.A., Ranheim, T., Leren, T.P. and Laerdahl, J.K. (2008) Investigations on the evolutionary conservation of PCSK9 reveal a functionally important protrusion. *FEBS J*, **275**, 4121-4133.
8. Hogarth, C.A., Roy, A. and Ebert, D.L. (2003) Genomic evidence for the absence of a functional cholesteryl ester transfer protein gene in mice and rats. *Comparative biochemistry and physiology. Part B, Biochemistry & molecular biology*, **135**, 219-229.
9. Ha, Y.C. and Barter, P.J. (1982) Differences in plasma cholesteryl ester transfer activity in sixteen vertebrate species. *Comparative biochemistry and physiology. B, Comparative biochemistry*, **71**, 265-269.
10. Guyard-Dangremont, V., Desrumaux, C., Gambert, P., Lallemant, C. and Lagrost, L. (1998) Phospholipid and cholesteryl ester transfer activities in plasma from 14 vertebrate species. Relation to atherogenesis susceptibility. *Comparative biochemistry and physiology. Part B, Biochemistry & molecular biology*, **120**, 517-525.
